# PAVOOC: designing CRISPR sgRNAs using 3D protein structures and functional domain annotations

**DOI:** 10.1093/bioinformatics/bty935

**Published:** 2018-11-16

**Authors:** Moritz Schaefer, Djork-Arné Clevert, Bertram Weiss, Andreas Steffen

**Affiliations:** 1 Computer Science, TU Berlin, D-10623 Berlin, Germany; 2 Bioinformatics, Bayer AG, D-13342 Berlin, Germany

## Abstract

**Summary:**

Single-guide RNAs (sgRNAs) targeting the same gene can significantly vary in terms of efficacy and specificity. PAVOOC (Prediction And Visualization of On- and Off-targets for CRISPR) is a web-based CRISPR sgRNA design tool that employs state of the art machine learning models to prioritize most effective candidate sgRNAs. In contrast to other tools, it maps sgRNAs to functional domains and protein structures and visualizes cut sites on corresponding protein crystal structures. Furthermore, PAVOOC supports homology-directed repair template generation for genome editing experiments and the visualization of the mutated amino acids in 3D.

**Availability and implementation:**

PAVOOC is available under https://pavooc.me and accessible using modern browsers (Chrome/Chromium recommended). The source code is hosted at github.com/moritzschaefer/pavooc under the *MIT License*. The backend, including data processing steps, and the frontend are implemented in Python 3 and ReactJS, respectively. All components run in a simple Docker environment.

**Supplementary information:**

Supplementary data are available at *Bioinformatics* online.

## 1 Introduction

The discovery of the CRISPR/Cas system (Cong *et al.*, 2013; Jinek *et al.*, 2012) was a breakthrough in the area of genome editing. An important application of CRISPR/Cas is to induce a targeted knockout (KO) of a gene of interest. Such KO experiments can help to study the essentiality of the targeted genes in given cellular contexts (e.g. a cancer cell line bearing certain genomic alterations) and ultimately support the validation of a new drug target (Moore, 2015). Shi *et al.* (2015) showed, that the effect of a CRISPR based KO can be boosted by targeting functionally relevant regions of a protein, as in these regions in-frame mutations (indels) are more likely to induce a significant effect than in non-functional regions. Another application of CRISPR/Cas is to precisely introduce missense mutations into a genome and study the resulting effects of the perturbations. In both applications single-guide RNAs (sgRNAs) are used to direct the Cas9 enzyme towards the genomic region of interest, such that the Cas9 can cut the DNA at the targeted position. For the genome editing experiments in addition a template sequence needs to be provided that contains the desired nucleotide sequence.

A number of tools have been published that facilitate and automate the design of sgRNAs for CRISPR KO experiments (Hough *et al.*, 2016; Listgarten *et al.*, 2018; Meier *et al.*, 2017; Stemmer *et al.*, 2015). In this application note, we present PAVOOC (Prediction And Visualization of On- and Off-targets for CRISPR)—a modern web application to support wet lab biologists in designing and selecting optimal sgRNAs and template sequences for KO and genome editing experiments using machine learning-based on- and off-target scoring, multi-attribute ranking, protein structure mapping of the cut sites and integration of cancer cell line data.

## 2 Materials and methods

PAVOOC is a web application that allows to design and visualize sgRNAs for gene KO and genome editing experiments. For KO experiments, a set of genes has to be provided (in form of symbols or Ensembl identifiers). PAVOOC then generates a table that contains a user-defined number of sgRNAs for each of these genes. These sgRNAs are prioritized based on the scoring function in Equation (1), which combines weighted on- and an off-target scores as well as whether the targeted regions lies within a protein domain. The on-target score is calculated using the Azimuth model, whereas the cutting frequency determination score is used to assess off-target effects (Doench *et al.*, 2016).

(1) $$\begin{array}{cc} ranking\_score & =0.6\cdot on\_target\_score\\ & \;+0.3\cdot (1-off\_target\_score)\\ & \;+0.1\cdot is\_in\_domain \end{array}$$

It is possible to further analyze and modify the sgRNA selection for a gene in a detail view (see Supplementary Fig. S1). The detail view consists of three synchronized panels: The ‘LineUp’ ranking table on the upper right, the protein structure view on the upper left and the sequence view on the bottom panel of the page. The LineUp (Gratzl *et al.*, 2013)-based sgRNA ranking table allows an individual adjustment of the weights for the on- and off-target scores in order to prioritize the sgRNAs accordingly. For each sgRNA, the LineUp table displays whether the targeted genomic region lies within a protein domain and whether the optionally selected cancer cell line contains a single nucleotide variation at that position. The sequence view on the bottom is based on Biodalliance (Down *et al.*, 2011) and shows the gene annotation, all targeted regions of the sgRNAs, protein domains and cancer cell line alteration data in order to support the tailored sgRNA design for a cell line under study. On the left side, available 3D protein structures from RCSB (Berman *et al.*, 2000) are shown and sgRNA-related cut sites are mapped and highlighted on the structure using the NGL viewer (Rose and Hildebrand, 2015). In this way, the user can assess the position of the Cas9 cut position on the protein structure and thus prioritize sgRNAs that are more likely to affect functionally relevant regions of a gene. Furthermore, when designing genome editing experiments, the structure view enables amino acid editing and displaying the designed alterations directly on the protein structure.

We integrated genomic sequence data from UCSC in version hg19 (Consortium *et al.*, 2001). The genomic annotations, including genes, transcripts and exons were taken from the GENCODE project (Harrow *et al.*, 2012). Cancer cell line alteration data was taken from the Cancer Cell Line Encyclopedia (Barretina *et al.*, 2012) (based on hg19). In order to facilitate the mapping between genomic and protein coordinates we used the canonical transcript from APPRIS (Rodriguez *et al.*, 2013) only. Exons which are not contained in that transcript are not considered in our application. SIFTS (Velankar *et al.*, 2012) mappings are used to derive genomic coordinates of PDB structures. A structured overview of our pipeline is shown in Supplementary Figure S2.

The data shown in the application is all pre-processed offline and stored in a non-relational database. Guide search and off-target scoring is performed using FlashFry (McKenna and Shendure, (2017).

## 3 Discussion

Our new tool PAVOOC provides a convenient means to design optimal sgRNAs for KO and genome editing experiments. A machine learning-based scoring system guides the user to select sgRNAs with possibly strong on- and low off-target effects. Through the integration of structural data, PAVOOC is able to display cut sites on corresponding protein crystal structures such that sgRNAs can be selected which cut in functionally relevant regions. Integration of cancer cell line data ensures that existing genomic alterations are considered during sgRNA selection. The tool was used internally to design a domain-targeting genome-wide sgRNA library.

PAVOOC is hosted on GitHub and is an actively maintained project. As such, it provides an open platform to build and integrate use cases of CRISPR that are not part of the current state. The PEP8 compliant Python code and the react.js-based frontend simplify the entry for developers. The application runs in a Docker environment which makes it easy to host the application on premise.

## Supplementary Material

bty935_Supplementary_Data
