# Supplementary material for: PAVOOC: designing CRISPR sgRNAs using 3D protein structures and functional domain annotations
Source: Bioinformatics. 2018 Nov 16;35(13):2309–10. doi: 10.1093/bioinformatics/bty935 (PMC6596878; doi:10.1093/bioinformatics/bty935)
Supplement: bty935_Supplementary_Data [file bty935_supplementary_data.pdf]

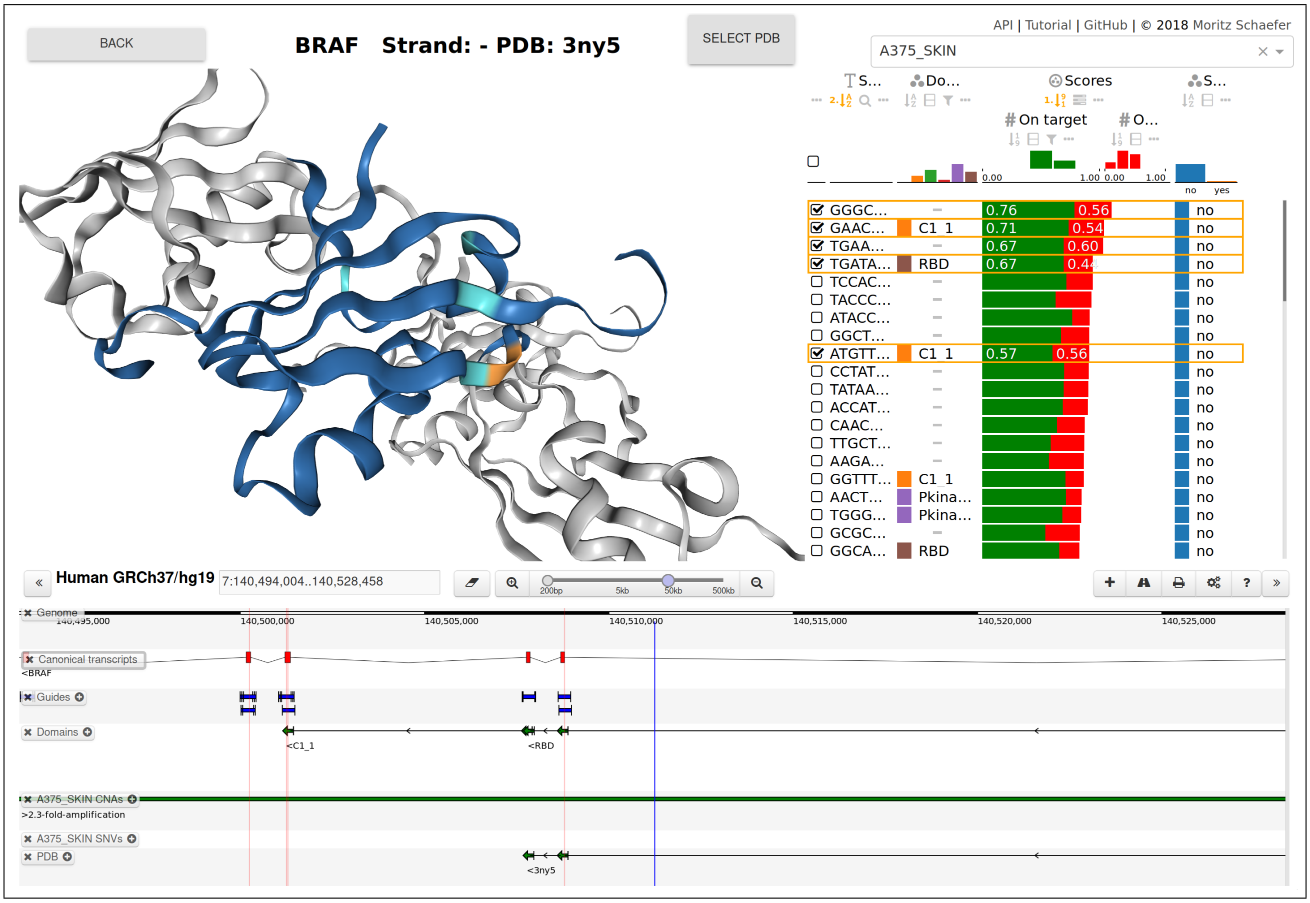

Supplementary Figure 1: Screenshot of the view to edit sgRNA selection, showing the LineUp ranking table and the protein structure view on the upper panels and the sequence view on the bottom panel.

The LineUp ranking table on the right highlights the five automatically selected sgRNAs. Note that the off-target score column (the red bars in the table) represent the inversion (i.e. $1-x$) of the CFD score such that lower CFD scores (which correspond to low off-target activity) contribute to a higher ranking of the associated sgRNA. The table is initially configured to order by on- and off-target scores (with ratio 65:35) and can be adjusted in different ways (e.g. the ratio between the on- and the off-target score can be altered by mouse dragging). The selected sgRNAs are indicated in the protein structure view such that the amino acid, affected by the sgRNA's cut position is highlighted in orange. Cut positions of other sgRNAs are highlighted in light blue. The protein structure consists of two separate chains from which only one is used for sgRNA mapping; the other one is grayed out. The sequence view on the bottom shows relevant information on the genome level and highlights the sgRNA targets with red bars. Using the "SELECT PDB" button, it is possible to show alternative PDB files. The input on the top right allows for the selection of cancer cell lines in order to investigate their possible effect on sgRNAs.

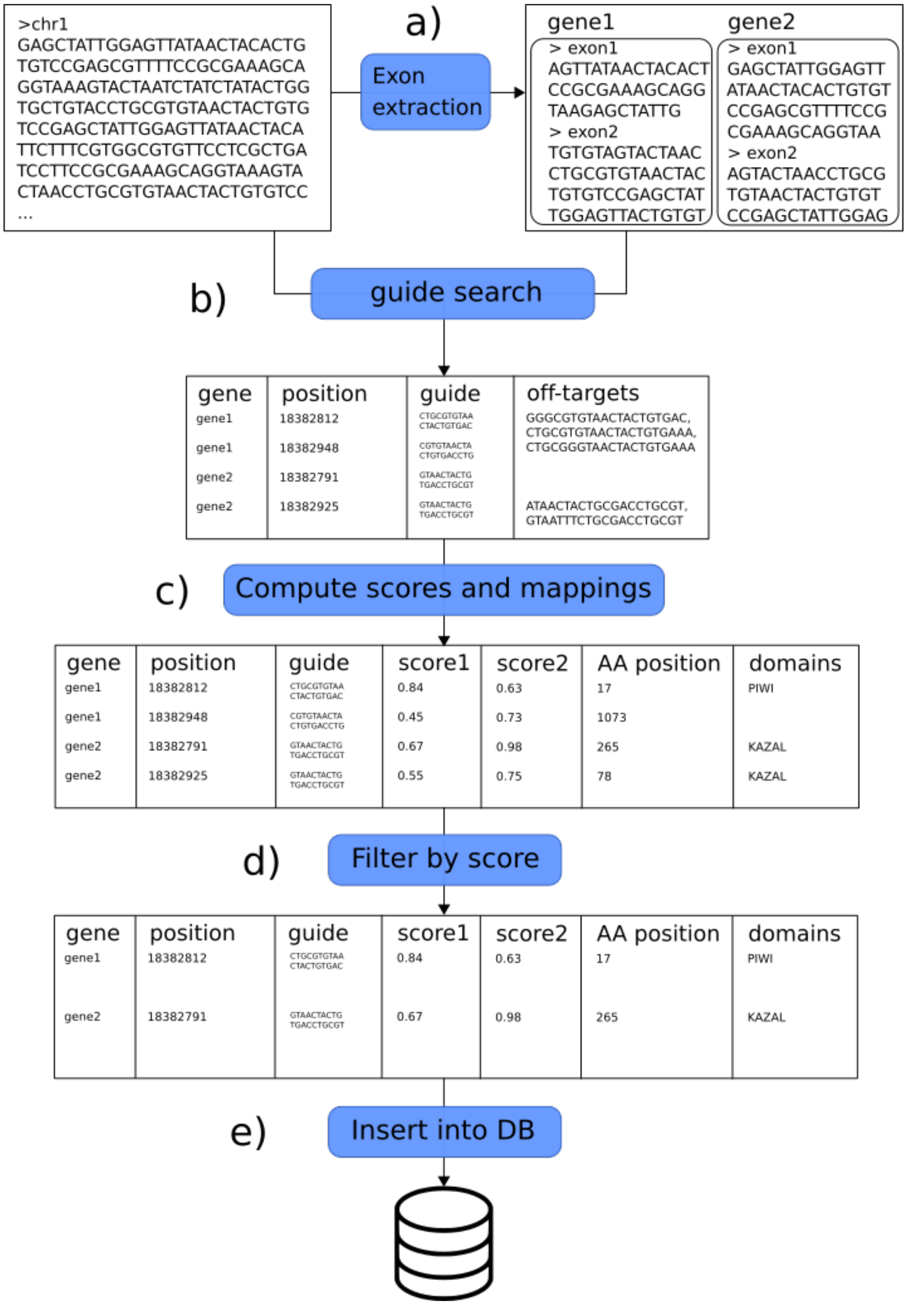

Supplementary Figure 2: The pipeline generating the data for the web application.

In step *a)* the positions of genes and corresponding exons are used from GENCODE (version 19) to extract their sequences from the human reference genome sequence (version hg19). The sequences are then stored in separate files, such that the search can be performed in the relevant regions: The exome (i.e. the part of the genome formed by exons). Searching the whole genome, including introns and non-coding regions would not be feasible in terms of processing time. In step *b)*, the extracted sequences are used to perform a guide search along

with potential off-targets using FlashFry. For the identified guides, mappings and efficacy/specificity scores are calculated in step *c)*. The scores are calculated using the default arguments for the Azimuth model (cited in the paper) and the CFD score (implemented in FlashFry). Guides containing sequences which interfere with CRISPR-Cas9 activity (i.e. guides starting with 'GGGGG' or containing one of 'TTTT', 'GGTCTC', 'GAGACC') or scoring less than 0.45 on the on-target efficacy are filtered in step *d)*. In step *e)*, guides along with the additional information computed in step *c)* are stored in a non-relational database, which forms the basis for the web application and the underlying API.
